# DNA Methylation-Based Age Prediction and Telomere Length Reveal an Accelerated Aging in Induced Sputum Cells Compared to Blood Leukocytes: A Pilot Study in COPD Patients

**DOI:** 10.3389/fmed.2021.690312

**Published:** 2021-07-23

**Authors:** Manuela Campisi, Filippo Liviero, Piero Maestrelli, Gabriella Guarnieri, Sofia Pavanello

**Affiliations:** ^1^Occupational Medicine, Department of Cardiac, Thoracic, and Vascular Sciences and Public Health, University Hospital of Padua, Padua, Italy; ^2^Respiratory Pathophysiology Unit, Department of Cardiac, Thoracic, and Vascular Sciences and Public Health, University Hospital of Padua, Padua, Italy

**Keywords:** DNA methylation age, age acceleration, induced sputum, chronic obstructive pulmonary disease, lung aging, telomere length

## Abstract

Aging is the predominant risk factor for most degenerative diseases, including chronic obstructive pulmonary disease (COPD). This process is however very heterogeneous. Defining the biological aging of individual tissues may contribute to better assess this risky process. In this study, we examined the biological age of induced sputum (IS) cells, and peripheral blood leukocytes in the same subject, and compared these to assess whether biological aging of blood leukocytes mirrors that of IS cells. Biological aging was assessed in 18 COPD patients (72.4 ± 7.7 years; 50% males). We explored mitotic and non-mitotic aging pathways, using telomere length (TL) and DNA methylation-based age prediction (DNAmAge) and age acceleration (AgeAcc) (i.e., difference between DNAmAge and chronological age). Data on demographics, life style and occupational exposure, lung function, and clinical and blood parameters were collected. DNAmAge (67.4 ± 5.80 vs. 61.6 ± 5.40 years; *p* = 0.0003), AgeAcc (−4.5 ± 5.02 vs. −10.8 ± 3.50 years; *p* = 0.0003), and TL attrition (1.05 ± 0.35 vs. 1.48 ± 0.21 T/S; *p* = 0.0341) are higher in IS cells than in blood leukocytes in the same patients. Blood leukocytes DNAmAge (*r* = 0.927245; *p* = 0.0026) and AgeAcc (*r* = 0.916445; *p* = 0.0037), but not TL, highly correlate with that of IS cells. Multiple regression analysis shows that both blood leukocytes DNAmAge and AgeAcc decrease (i.e., younger) in patients with FEV_1_% enhancement (*p* = 0.0254 and *p* = 0.0296) and combined inhaled corticosteroid (ICS) therapy (*p* = 0.0494 and *p* = 0.0553). In conclusion, new findings from our work reveal a differential aging in the context of COPD, by a direct quantitative comparison of cell aging in the airway with that in the more accessible peripheral blood leukocytes, providing additional knowledge which could offer a potential translation into the disease management.

## Introduction

The aging of the population, also called the “gray” revolution, is undoubtedly an emerging social and public health problem. Aging is an individual and very complex process characterized by a progressive decline in the body's ability to respond to internal and/or external stressors (1). This deterioration is the primary risk factor for major human degenerative pathologies, including cancer and cardiovascular, neurodegenerative, and respiratory diseases (1). Chronic obstructive pulmonary disease (COPD) is one of the major causes of chronic morbidity (2) and the third leading cause of death worldwide^1^ which is projected to rise (3) because of the aging population. Some individuals, however, have a physiological senescence that is faster than that of others (1). In fact, not all individuals grow older in the same way (4); consequently, chronological age may not be a reliable indicator of physiological decline (5).

Exploring the aging process, defining measurable estimates of “biological aging” (in contrast to chronological aging), has become a major initiative in medical research (6). Two “pillars of aging” have been proposed as the most promising early indicators for aging (6), i.e., telomere shortening and epigenetic alterations in DNA, which primary cause damage to cellular functions. Telomeres act as a mitotic clock that, by reducing itself at each cell division, leads to cellular senescence (replicative senescence) or cell death. A powerful emerging marker of non-mitotic cellular aging is the epigenetic age often defined as DNA methylation age (DNAmAge) (7, 8). DNAmAge in human (9–11) is assessed from methylation at a species-specific subset of cytosine–guanine dyads (CpG), and it is strongly correlated with chronological age (9–13). Development of epigenetic predictors has addressed to an “epigenetic clock” theory of aging, according to which the difference between DNAmAge and chronological age is defined as “age acceleration” (AgeAcc) (14), which is indicative of altered biological functions (8) and elevated risk for morbidity and mortality (15).

COPD shows striking lung aging-associated features including the reduction of function, pulmonary inflammation (2), and progressive airway obstruction (16, 17). Understanding of age-related pathomechanisms associated with COPD is decisive to also uncovering novel strategies for disease treatment. Smoking is the primary cause for COPD worldwide (18), and in developing countries, COPD also arises as a result of exposure to household air pollution (18, 19). The mechanisms linking tobacco smoke and other air pollutants with COPD have not yet been fully elucidated. One main working hypothesis is that air pollutants, especially the particulate matter (PM) of aerodynamic diameter of 2.5 μm or less, can deeply penetrate into the lung, deposit in the alveolar area, and locally trigger oxidative stress and inflammatory response (20). The pulmonary local oxidative–inflammatory reaction damages lung tissue, leading to structural pathological changes such as lung parenchyma destruction (emphysema), fibrosis of peripheral airways, and an increase in mucus-producing cells, with a consequent airflow limitation, which are all signs of an accelerated aging of the lung (20). This local oxidative–inflammatory reaction leads to a subsequent systemic inflammation (21). Several blood inflammatory markers, including C-reactive protein (CRP) (22), interleukin (IL)-6 (23), and blood leukocytes, are all found altered in COPD patients (24). Altered inflammatory responses and induced oxidative stress are two key mechanisms accelerating biological aging detected by early signs of cellular aging, including alterations in telomere length (TL) (25) and DNA methylation (26). Shorter TL in blood leukocytes has been described from patients with COPD (27) and in a meta-analysis of 14 studies (28). One recent study from two independent longitudinal large cohort studies relates DNAmAge in blood leukocytes to COPD incidence (29). However, no epigenetic age estimation was performed in the target organ of the disease, i.e., the lung.

While lung tissue is not routinely accessible, sputum induction represents a validated noninvasive method of lower respiratory tract sampling for analysis of cell components in the airways lumen and fluid-phase constituents (30). It has been successfully applied for assessing disease severity and progression in COPD, producing reliable results comparable to biopsy and bronchoalveolar lavage (31).

This study has two main objectives:

i) To determine the biological age of the induced sputum (IS) cells and peripheral blood leukocytes, by measuring the mitotic age (TL) and non-mitotic epigenetic age (DNAmAge).
ii) To compare blood leukocytes and IS cells in order to assess the reliability of blood leukocytes as an accurate indicator of lower respiratory tract biological age.

To these aims, COPD patients were examined as a positive paradigm of lung aging, taking into consideration their demographic data, life style and occupational exposure, lung function, and clinical and blood parameters.

## Materials and Methods

### Study Design

The present study includes *n* = 18 moderate patients with COPD, diagnosed according to GOLD guidelines (32), enrolled at the ambulatory of Respiratory Physiopathology Ward – Occupational Medicine, Department of Cardio-Vascular-Thoracic Science and Public Health, University of Padova. The local Ethics Committee - University of Padova approved the study protocols (3843/AO/16 and 3054/AO/14). The recruiting of COPD patients was carried out between September 2018 and September 2019. The inclusion criteria for the study participation were post-bronchodilator forced expiratory volume in a 1-s (FEV_1_)/forced vital capacity (FVC) ratio of <70% on spirometry and no acute exacerbation for at least 6 weeks. All patients were informed of the purpose of the study by trained interviewers and asked to sign an informed consent form. The study was conducted in accordance with the Declaration of Helsinki. Participants were interviewed with structured questionnaires to collect information regarding demographic data (age, gender), age of parents at birth and educational level (years), smoking history and pack-years, alcohol intake in the last 12 months and habitual alcohol consumption measured as unit of drink/day (1 unit = 10–12 g alcohol intake), environmental exposure (diet, indoor, home, traffic, outdoor), physical activity (IPAQ score), clinical determinants (e.g., leukocytes, blood red cells, hemoglobin, glycemia, CRP), medical history, and therapy. Therapy information included inhalation device types available, such as metered-dose inhalers (MDIs), dry-powder inhalers (DPIs), and soft mist inhalers (SMIs). Specifically, therapy has been prescribed according to the GOLD guidelines (32), in which from 2011 the assessment approach acknowledges the limitations of FEV_1_ in making treatment decisions for individualized patient care and highlights the importance of patient symptoms and exacerbation risk in guiding therapies in COPD, the “ABCD” assessment tool. All patients underwent a physical examination, and lung function was assessed by spirometry recording forced expiratory volume in 1 s (FEV_1_), FVC, vital capacity (VC), total lung capacity (TLC), residual volume (RV), and FEV_1_/VC ratio also defined as Tiffeneau index. For each patient, blood samples were collected in vacutainer K3EDTA tubes and PAXgene tubes, for basic biochemistry, TL determination, and DNAmAge assessment. A plasma sample was also collected and stored in a freezer at −80°C for further investigations. During medical examination, the procedure of sputum induction was carried out for each patient to collect a sample of airways cells on which to analyze TL and DNAmAge.

### IS Procedure and Spirometry

All lung function measurements were measured using a spirometer (MasterScreen PFT, PRO, Viasys Sanità, Firenze, Italy) according to the guidelines/recommendations of the American Thoracic Society/European Respiratory Society (ATS/ERS) (33). FEV_1_ was used as the primary variable of lung function which was measured both before and 10 min after the use of the post-bronchodilator. FEV_1_ was expressed as liters and as a percentage of the predicted normal value (FEV_1_%) according to reference values based on age, height, weight, sex, and race for each subject using the European Community for Steel and Coal as reference values (34). These values obtained are in turn used as reference standards/assessments for consecutive spirometries performed during the IS standard procedure (35). Nebulized sterile saline solutions (hypertonic at 3 and 4%) were consecutively administered using a nebulizer [UltraNeb, DeVilbiss, Desio (MB), Italy] with an output flow of ~1 ml∙min in four sequential 5-min inhalation periods. Since saline inhalation may cause bronchoconstriction, after each inhalation period FEV_1_ was measured for the detection and monitoring of lung function during the process, stopping the procedure when FEV_1_ decreased over 20% compared with that of post-salbutamol baseline. During the procedure, the patient was asked to cough and expectorate. Once collected, IS was processed according to a standard technique (35). The weight of the selected sputum plugs was recorded and the sample was diluted with a volume of phosphate-buffered solution (PBS) and 0.1% dithiothreitol (DTT) equal to 4:1 of selected plugs. After filtration with a nylon mesh (52–56 μm), the sample was centrifuged (3,000 rpm for 3 min) to separate cells and supernatant. The cell pellet was resuspended in 1 ml of PBS. The cells were stained for viability assessment using an equal volume (10 μl) of both sample and Trypan Blue. The cell concentration was adjusted to obtain a final concentration of ~300,000 cells/ml. The cells were cytocentrifuged (Cytospin, Shandon Scientific, Milano, Italy) at 450 rpm for 6 min, onto glass slides treated with aptex (3-aminopropyltriethoxysilane) according to a standard method (36) and stained with Diff-Quik (Dade Behring, Milano, Italy). The differential cell count in IS was measured counting 400 nucleated cells per each of two slides stained reporting the percentage of eosinophils, neutrophils, macrophages, lymphocytes, and bronchial epithelial cells. The IS sample is considered acceptable and adequate if the percentage of squamous cells is <20% of the total cells, warranting the reproducibility of cell counts.

### DNA Extraction From Blood Samples

DNA was extracted from whole blood using the QIAamp DNA Mini Kit (Qiagen, Milano, Italy) on a QIAcube System (Qiagen, Milano, Italy) for automated high-throughput DNA purification, according to a customized protocol as previously described (13). In particular, 400 μl of whole blood from each sample was processed for DNA extraction. DNA was quantified and checked for quality using QIAxpert Quantification System (Qiagen, Milano, Italy).

### DNA Extraction From IS Samples

Once the IS sample was collected to analyze the differential cell count, a second aliquot of the same sample was processed as indicated in the protocol reported in IS procedure and spirometry up to the centrifugation step (3,000 rpm for 3 min) to separate cells and supernatant. The cell pellet was then resuspended in 180 μl of PBS. DNA extraction was performed on the automated QIAcube System (Qiagen, Milano, Italy) using QIAamp DNA Mini Kit (Qiagen, Milano, Italy) according to a customized protocol developed for highly viscous samples. After extraction, DNA was quantified and checked for quality using the QIAxpert Quantification System (Qiagen, Milano, Italy).

### TL Analysis

TL was measured after DNA extraction from both whole blood and IS samples, by using quantitative real-time PCR as previously described (37, 38). This assay measures relative TL in genomic DNA by determining the ratio of telomere repeat copy number (T) to a single nuclear copy gene (S) in experimental samples relative to the T/S ratio of a reference pooled sample. The single-copy gene used was human (beta) globin (hbg). The PCR runs were conducted in triplicate on a StepOnePlus Real-Time PCR System (Applied Biosystems, Milano, Italy), and the average of the three T/S ratio measurements was used in the statistical analyses. Details of TL analysis are reported in the Supplementary Materials.

### DNAmAge and AgeAcc Analyses

DNAmAge was determined by analyzing the methylation levels from selected markers using the bisulfite conversion and Pyrosequencing® methodology as previously reported (12, 13). This method is based on determination of the methylation level of a set of five markers (ELOVL2, C1orf132, KLF14, TRIM59, and FHL2) in genomic DNA, as described by Zbieć-Piekarska et al. (11) with some modifications based on the fact that the method was almost completely automated using the PyroMark Q48 Autoprep (Qiagen, Milano, Italy) (12, 13). AgeAcc was calculated as the difference between the detected DNAmAge of IS cells and blood leukocytes and the chronological age of patients. Details of DNAmAge analysis are reported in the Supplementary Materials.

### Statistical Analysis

Statistical analyses were performed with StastDirects software. Data are expressed as mean ± SD or number and percentage. The diversity among the two groups of patients split per therapy (dual therapy and triple therapy with combined inhaled corticosteroid (ICS) assumption) was appraised with the Mann–Whitney *U*-test and chi-square test, respectively. Levels of TL, DNAmAge, and AgeAcc in IS cells, and blood leukocytes, of the same patient, were compared by the (two-tailed) paired *T*-test. Comparison between all samples in the two groups was also made using the Mann–Whitney *U*-test. Simple linear regression was evaluated in order to provide a measure of the strength of dependence between two variables. Correlations between age, leukocytes, differential cell count, and cigarette smoking (pack years) (independent variables) on blood leukocytes DNAmAge, AgeAcc, and TL measures (dependent variables) were evaluated by simple linear regression models. Lastly, age and differential cell counts of IS, including % macrophages, % neutrophils, and % eosinophils (independent variables), were related by simple linear regression to DNAmAge, AgeAcc, and TL, respectively. The influence of age, gender (female), ICS therapy, leukocytes (10^3^/ml) (model a), and neutrophils (10^3^/ml) (model b) and FEV_1_% as indicator of lung function on blood leukocytes TL, DNAmAge, and AgeAcc was appraised by multiple linear regression analyses. Lastly, the influence of age, gender (female), ICS therapy, type of inhalers, neutrophil percentage (%), and FEV_1_% on IS cell TL, DNAmAge, and AgeAcc was appraised by multiple linear regression analyses. Results were considered significant when a *p*-value of < 0.05 was obtained.

### Sample Size Estimation

Sample size estimation for a paired *T*-test was applied to calculate the sample size. The calculation was computed through a STATA command by specifying a mean difference = 0.43, 5.8, and 6.0, and standard deviation of differences 0.14, 0.40, and 1.52, respectively, for IS cells vs. blood leukocytes TL, DNAmAge, and AgeAcc, respectively. The group size to obtain statistical significance with α (two-tailed) = 0.05 and β = 0.20 was estimated to be *n* = 4, *n* = 2, and *n* = 3 subjects for TL, DNAmAge, and AgeAcc, respectively.

## Results

### Characteristics of the Study Population

The characteristics of the study subjects are reported in Tables 1, 2. Interval variables (mean ± SD) of all COPD patients (*n* = 18) with a long-acting β2 agonist/long-acting muscarinic antagonist (LABA/LAMA) (*n* = 9) and with combined ICS therapy and LABA/LAMA administration (*n* = 9), also defined as dual and triple therapy, respectively, are shown in Table 1. The comparison of two groups (Mann–Whitney *U*-test) indicates that patients in LABA/LAMA therapy present higher values of FEV_1_ (*p* = 0.0003), FEV_1_% (*p* < 0.0001), FVC (*p* = 0.0003), VC (*p* = 0.0003), and TLC (*p* = 0.0008) and also a lower systolic pressure (*p* = 0.036), than those with ICS therapy and LABA/LAMA. No difference in the other parameters is observed. In particular, functional data of COPD patients including FEV_1_% are categorized according to inhalation therapy, dual therapy, or triple therapy. Furthermore, in our study population, the majority of patients in dual therapy used single or combinations of DPI inhalers (*n* = 6); only few cases used MDI (*n* = 1) and SMI inhalers (*n* = 2), alone. COPD patients in triple therapy used predominantly single or combinations of DPI inhalers (*n* = 6), *n* = 1 MDI with DPI devices, *n* = 1 SMI with DPI devices, and *n* = 1 combined SMI and MDI devices. Furthermore, patients in triple therapy, which are those with more symptoms and more exacerbations, presented also lower functional average values including FEV_1_%, compared to patients in dual therapy. Table 2 shows the number and percentage of categorical variables in the same groups. All characteristics are equally distributed among the two groups with dual and triple therapy, including in particular smoking history, the main risk factor for COPD insurgence (chi-square test *p* = not significant).

**Table 1:** Interval variables in COPD patients with long-acting β2 agonist (LABA)/long-acting muscarinic antagonist (LAMA) and inhaled corticosteroid (ICS)/LABA/LAMA treatments (mean ± SD) and *p*-values of the Mann–Whitney test comparing the two groups.

**Variables**	**All patients *n* = 18**	**Laba/Lama *n* = 9**	**ICS/Laba/Lama *n* = 9**	***p*-value**
Age (years)	72.4 ± 7.7	71.1 ± 9.0	73.7 ± 6.4	0.502
Education (years)	9.9 ± 4.4	9.2 ± 4.5	10.7 ± 4.4	0.504
Body mass index (kg/m^2^)	27.62 ± 4.5	27.1 ± 5.1	28.0 ± 4.0	0.561
Systolic pressure (mm Hg)	133.6 ± 12.5	128.3 ± 12.7	138.9 ± 10.2	**0.036**
Diastolic pressure (mm Hg)	81.4 ± 6.6	78.9 ± 6.9	83.9 ± 5.5	0.154
Mother age (years)	30.5 ± 5.9	29.4 ± 7.5	31.4 ± 4.6	0.319
Father age (years)	35.3 ± 6.8	32.8 ± 6.3	37.5 ± 6.8	0.236
Pack years [(cigarettes/20) × years]	33.4 ± 17.6	33.5 ± 15.5	33.3 ± 20.4	0.983
Drinking (age at start, years)	14.6 ± 10.4	15.3 ± 10.5	14.1 ± 11.0	0.910
Alcohol (daily intake last year)	0.6 ± 0.6	0.9 ± 0.7	0.3 ± 0.4	0.123
Sport (IPAQ score)	191.7 ± 376.8	363 ± 483.4	20 ± 42.4	0.077
Leukocytes (10^3^/ml)	6.5 ± 1.9	5.9 ± 1.5	7.0 ± 2.3	0.385
Blood red cells (10^3^/ml)	4.6 ± 0.4	4.6 ± 0.5	4.6 ± 0.4	0.983
Hemoglobin (g/dl)	13.2 ± 1.8	13.7 ± 2.1	13.7 ± 1.4	0.373
Platelet count (10^3^/ml)	231.9 ± 49.5	231.2 ± 40.2	232.5 ± 59.9	0.843
Neutrophils (10^3^/ml)	3.99 ± 1.4	3.4 ± 0.9	4.5 ± 1.6	0.094
Lymphocytes (10^3^/ml)	1.6 ± 0.6	1.5 ± 0.5	1.6 ± 0.7	0.981
Monocytes (10^3^/ml)	0.6 ± 0.2	0.5 ± 0.1	0.6 ± 0.2	0.351
Eosinophils (10^3^/ml)	0.2 ± 0.3	0.3 ± 0.4	0.1 ± 0.1	0.979
Basophils (10^3^/ml)	0.04 ± 0.04	0.03 ± 0.02	0.05 ± 0.05	0.493
Glycemia (mg/dl)	97.5 ± 35.9	110.3 ± 32.6	80.9 ± 35.3	0.238
C-reactive protein (mg/ml)	3.2 ± 3.2	2.7 ± 1.9	3.7 ± 4.4	0.983
FEV_1_ l/s	1.5 ± 0.6	1.9 ± 0.5	0.99 ± 0.3	**0.0003**
FEV_1_%	63.1 ± 16.6	77.1 ± 9.1	49.1 ± 7.9	** <0.0001**
FVC l/s	2.4 ± 0.9	3.2 ± 0.8	1.7 ± 0.4	**0.0003**
VC	2.5 ± 0.9	3.2 ± 0.8	1.7 ± 0.4	**0.0003**
TLC	4.9 ± 1.3	5.8 ± 1.3	3.96 ± 0.5	**0.0008**
RV	2.4 ±0.6	2.6 ± 0.7	2.2 ± 0.4	0.474
FEV_1_/VC%	58.6 ± 7	60.1 ± 8.0	57.0 ± 6.8	0.489

*Bold character is displayed only for significant values*.

**Table 2:** Distribution of categorical variables in COPD patients with *p*-values of the chi-square test comparing the two groups.

**Variables**	**Classes**	**All patients**	**Laba/Lama**	**ICS/Laba/Lama**	***p*-value**
		**N (%)**	**N (%)**	**N (%)**	
Sex ^@^	Males	9 (50)	6 (67)	3 (33)	0.202
Smoking	Nonsmokers	1 (5)	0 (0)	1 (11)	0.500
	Ex-smokers	15 (83)	8 (89)	7 (78)	0.603
	Smokers	2 (11)	1 (11)	1 (11)	0.999
Drink ^@^	Drinkers	13 (72)	7 (78)	6 (67)	0.750
Binge	None	0 (0)	0 (0)	0 (0)	NA
Charlson index	≤ 1	1 (5)	1 (11)	0 (0)	0.500
	≥ 2 ≤ 4	10 (55)	4 (44)	6 (67)	0.395
	≥5	7 (39)	4 (44)	3 (33)	0.667

### Biological Age of the IS Cells and Blood Leukocytes Determined by DNAmAge, AgeAcc, and TL

**Figure 1:**
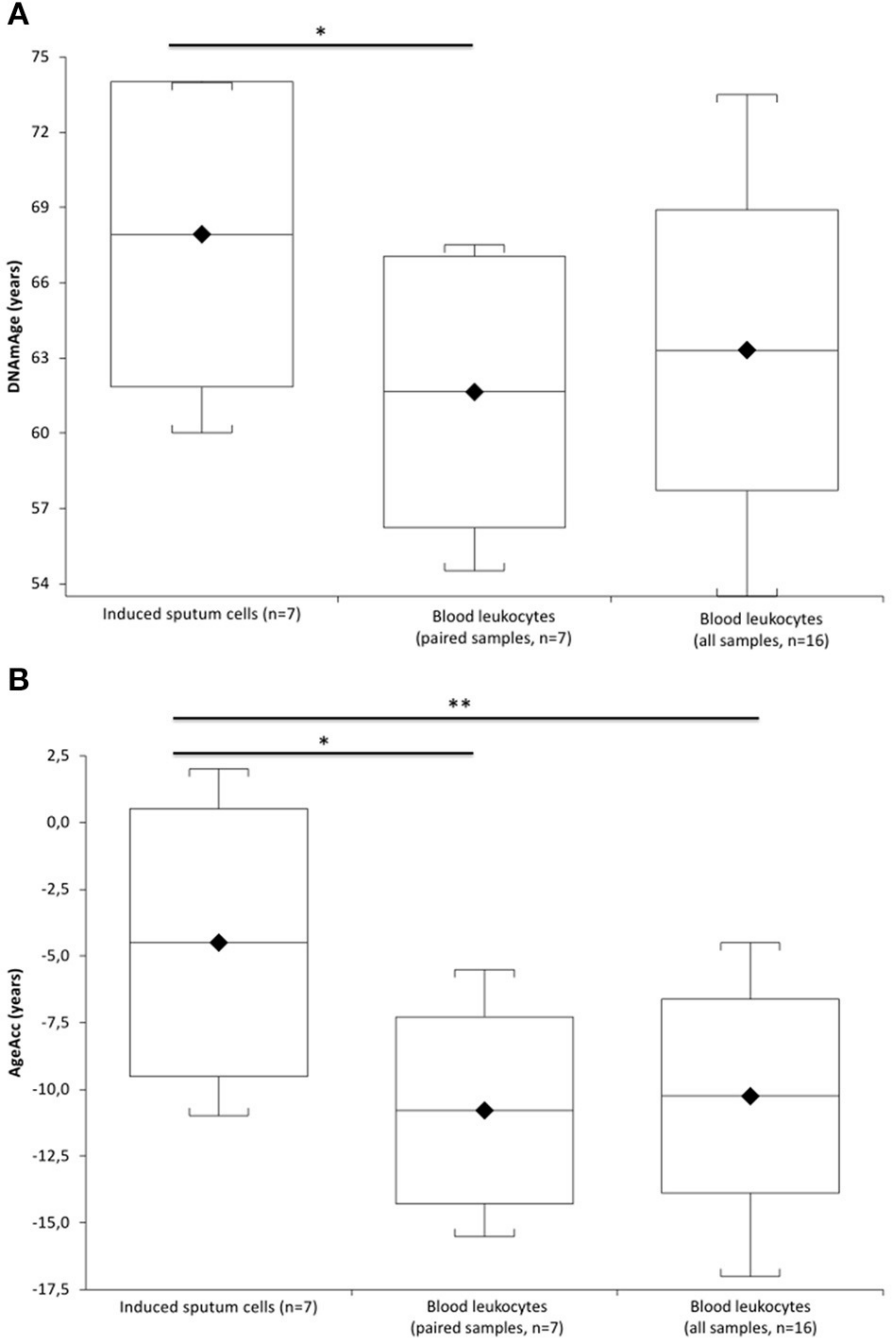
DNAmAge and AgeAcc of the induced sputum cells and blood leukocytes in COPD patients. In **(A)**, box plots show levels of DNAmAge in induced sputum cells (*n* = 7) and in paired blood leukocytes of the same COPD patient (*n* = 7), and in blood leukocyte samples of all COPD patients (*n* = 16). In box plots, the boundary of the box closest to the x-axis indicates the 25th percentile, the line within the box marks the mean, and the boundary of the box farthest from the x-axis indicates the 75th percentile. Whiskers (error bars) above and below the box indicate the 95 and 5th percentiles. The horizontal bar with asterisk indicates the significant comparison between induced sputum cells (*n* = 7) and paired blood leukocytes DNAmAge of the same patient (*n* = 7) (*Paired *t*-test: mean 67.4 ± 5.80 years vs. mean 61.6 ± 5.40 years; *p* = 0.0003). In contrast, the comparison between the DNAmAge of the induced sputum cells (*n* = 7) and all blood leukocytes (*n* = 16) is not significant (Mann–Whitney *U*-test: mean 67.4 ± 5.80 years vs. mean 63.3 ± 5.60 years; *p* = 0.1589). In **(B)**, box plots show levels of AgeAcc in induced sputum cells (*n* = 7) and in paired blood leukocytes of the same COPD patient (*n* = 7), and in blood leukocytes samples of all COPD patients (*n* = 16). In box plots, the boundary of the box closest to the x-axis indicates the 25th percentile, the line within the box marks the mean, and the boundary of the box farthest from the x-axis indicates the 75th percentile. Whiskers (error bars) above and below the box indicate the 95th and 5th percentiles. The horizontal bar with an asterisk indicates the significant comparison between induced sputum cells (*n* = 7) and paired blood leukocytes AgeAcc of the same patient (*Paired *t*-test (*n* = 7): mean −4.5 ± 5.02 years vs. mean −10.8 ± 3.50 years; *p* = 0.0003]. The upper longer horizontal bar with two asterisks indicates the significant comparison between AgeAcc of the induced sputum cells (*n* = 7) and blood leukocytes in all patients (*n* = 16) (**Mann–Whitney *U*-test: mean −4.5 ± 5.02 years vs. mean −10.3 ± 3.63 years; *p* = 0.0156].

**Figure 2:**
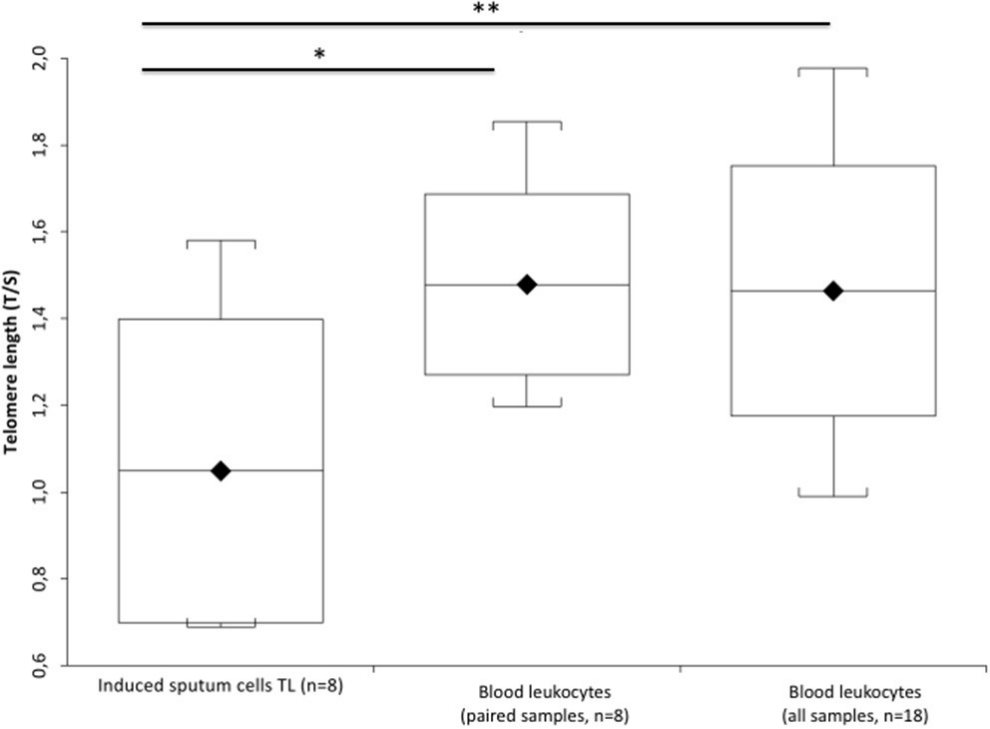
TL of the induced sputum cells and blood leukocytes in COPD patients. Box plots show levels of TL in induced sputum cells (*n* = 8) and in paired blood leukocytes of the same patient (*n* = 8), and blood leukocyte samples of all patients (*n* = 18). In box plots, the boundary of the box closest to the x-axis indicates the 25th percentile, the line within the box marks the mean, and the boundary of the box farthest from the x-axis indicates the 75th percentile. Whiskers (error bars) above and below the box indicate the 95 and 5th percentiles. The horizontal bar with one asterisk indicates the significant comparison between induced sputum cells (*n* = 8) and paired blood leukocyte TL of the same patient (*n* = 8) (*Paired *t*-test: mean 1.05 ± 0.35 T/S vs. mean 1.48 ± 0.21 T/S; *p* = 0.0341). The upper longer horizontal bar with two asterisks indicates the significant comparison between TL of the induced sputum cells (*n* = 8) and blood leukocytes in all patients (*n* = 18) (**MannWhitney *U*-test: mean 1.05 ± 0.35 T/S vs. mean 1.47 ± 0.26 T/S; *p* = 0.0133).

Figure 1A (Supplementary Table 2) shows that IS cell DNAmAge is older (mean 6.3 ± 2.08 years) than blood leukocyte DNAmAge in the same patient (*n* = 7, paired *t*-test mean 67.4 ± 5.80 years vs. mean 61.6 ± 5.40 years; *p* = 0.0003). IS cell AgeAcc (Figure 1B; Supplementary Table 2) is also extremely enhanced compared to that of blood leukocytes in the same patient (*n* = 7, paired *t*-test mean −4.5 ± 5.02 years vs. mean −10.8 ± 3.50 years; *p* = 0.0003) and in all patients (*n* = 16, Mann–Whitney *U*-test: mean −4.5 ± 5.02 years vs. mean −10.3 ± 3.63 years; *p* = 0.0156). Likewise, Figure 2 (Supplementary Table 3) reports that IS cell TL mean is shorter than that of blood leukocytes in the same patient (*n* = 8, paired *t*-test: mean 1.05 ± 0.35 T/S vs. mean 1.48 ± 0.21 T/S; *p* = 0.0341) and in all patients (*n* = 18, Mann–Whitney *U*-test: mean 1.05 ± 0.35 T/S vs. mean 1.47 ± 0.26 T/S; *p* = 0.0133). The discordance, between number of samples displayed in Figure 1 (Supplementary Table 2) and Figure 2 (Supplementary Table 3), has to be ascribed to the insufficient amount of DNA available to perform the analysis of DNAmAge for three samples that was instead enough to analyze TL in all blood and IS samples.

**Figure 3:**
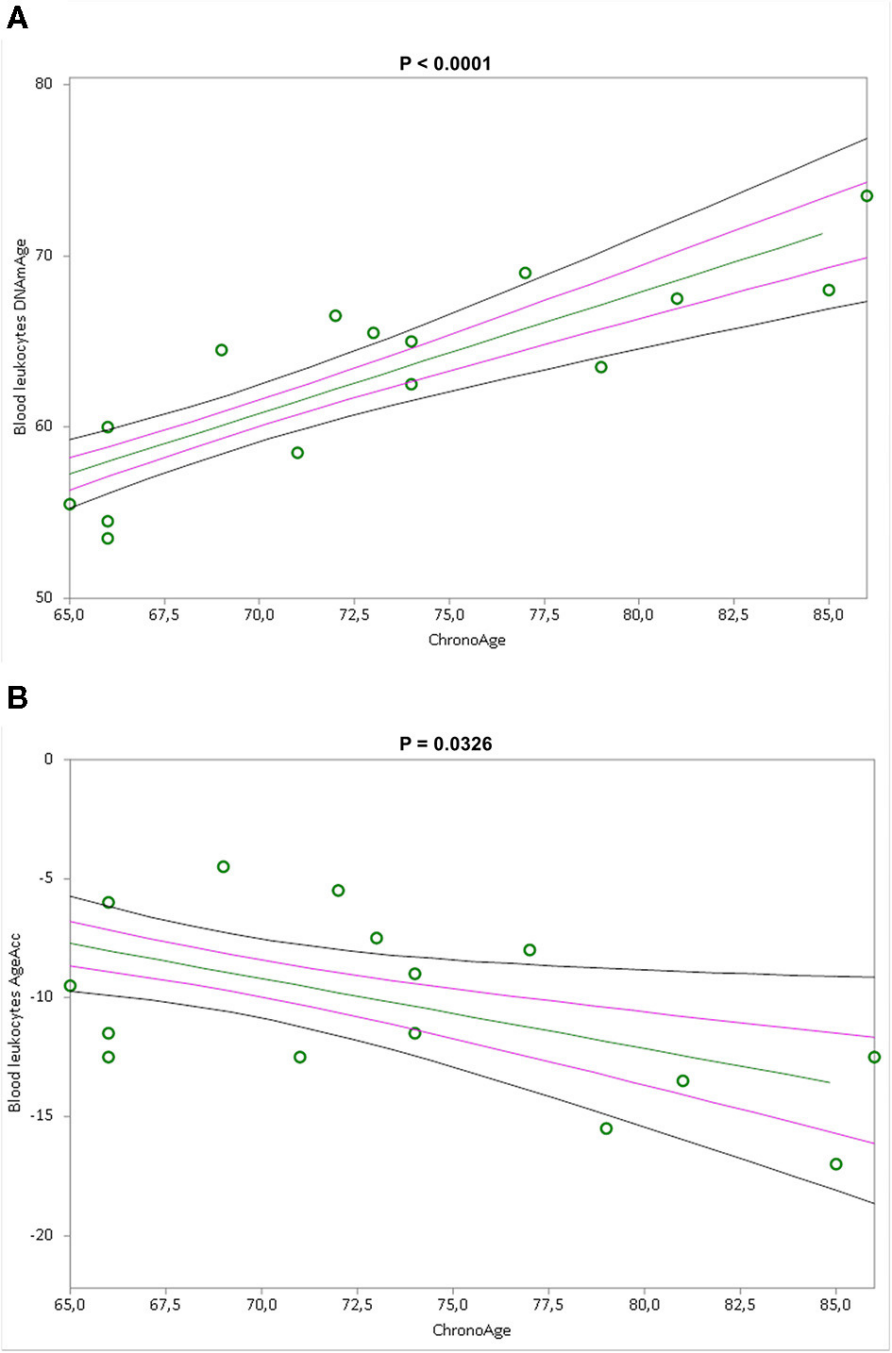
Correlation curves between blood leukocytes DNAmAge **(A)** or AgeAcc **(B)** and chronological age of *n* = 16 COPD patients. In **(A)**, a simple linear regression plot shows the correlation between blood leukocyte DNAmAge and chronological age [correlation coefficient (r) = 0.836142; two-sided *p* < 0.0001], while in **(B)**, simple linear regression linear regression plot showing the correlation between blood leukocyte AgeAcc and chronological age [correlation coefficient (r) = −0.53542; two-sided *p* = 0.0326]. Mean, standard error (SE), and 95% coefficient intervals (CI) are represented as green, pink, and black lines, respectively.

Simple linear regression analyses show that blood leukocytes DNAmAge and AgeAcc were highly associated with chronological age (*p* < 0.0001 and *p* = 0.0326 in Figures 3A,B), but not the IS cell DNAmAge and AgeAcc (*p* = 0.1104 and *p* = 0.3717, in Supplementary Figures 1A,B) as well as IS cell and blood leukocyte TL (*p* = 0.460 and *p* = 0.2705, in Supplementary Figures 1C,D). Furthermore, blood leukocyte DNAmAge, AgeAcc, and TL measures were not correlated with leukocytes and different blood cell counts (Supplementary Table 4) and to cigarette smoking (pack years) (Supplementary Table 5). On the other hand, differential cell counts of IS (Supplementary Table 6), in particular macrophage % negatively (*p* = 0.033) and neutrophil % positively (*p* = 0.011), but not eosinophil % (*p* = 0.1239), are related to DNAmAge, while IS cell AgeAcc and TL are not related to cell counts. Furthermore, as expected, neutrophils represent the higher cell type of IS (neutrophils: 68.06 ± 27.03 %, macrophages: 25.19 ± 16.81 % and eosinophils: 6.75 ± 15.70 %). We want also point out that no active smokers were in the IS samples.

### Correlation Between Blood Leukocytes and IS Cells Biological Age

**Figure 4:**
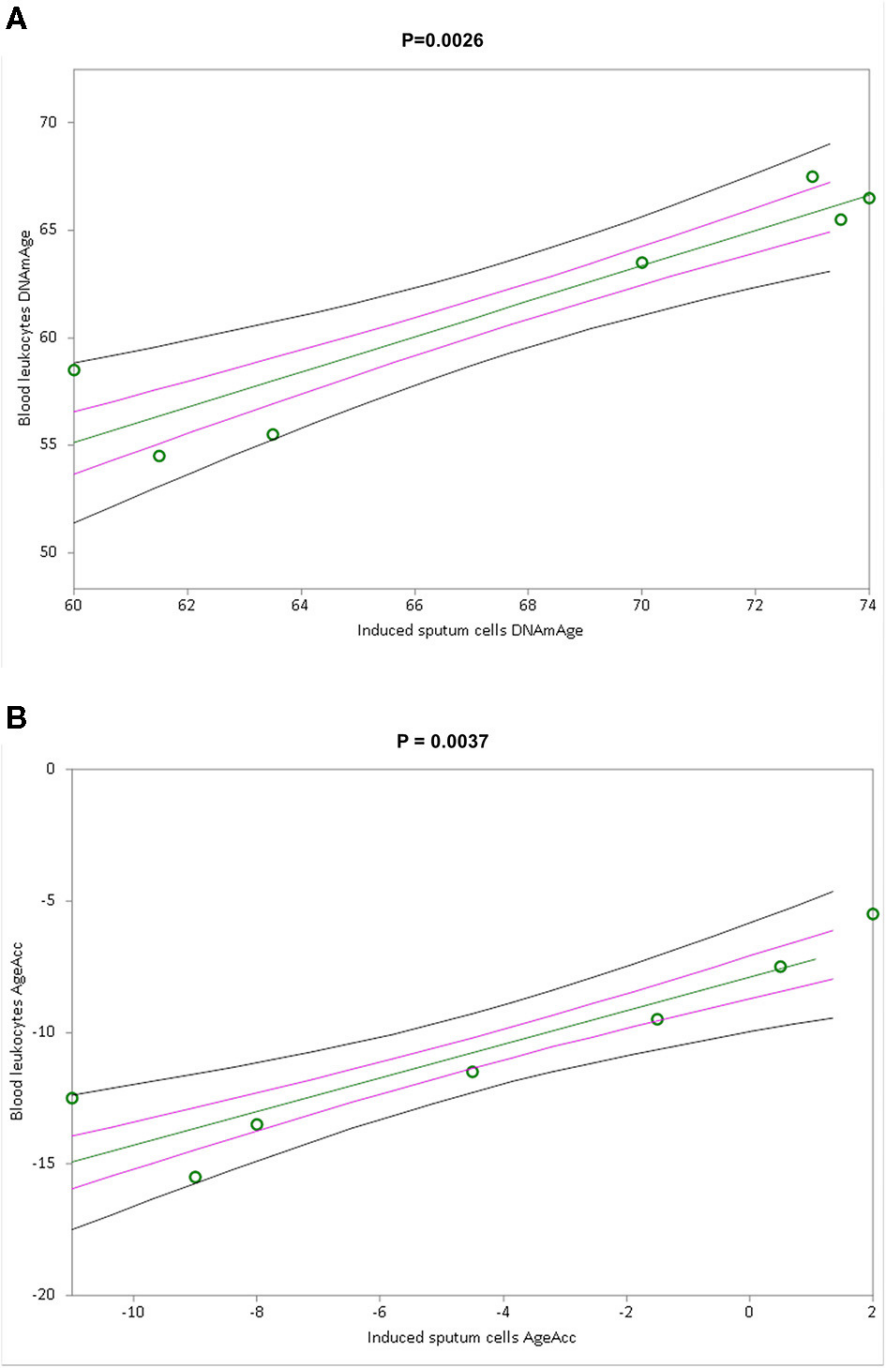
Correlation curves between blood leukocytes and induced sputum cells DNAmAge **(A)** or AgeAcc **(B)** of *n* = 7 COPD patients. In **(A)**, a simple linear regression plot shows the correlation between blood leukocytes and induced sputum cell DNAmAge [correlation coefficient (r) = 0.927245; two-sided *p* = 0.0026], whereas in **(B)**, a simple linear regression plot shows the correlation between blood leukocytes and induced sputum cells AgeAcc [correlation coefficient (r) = 0.916445; two-sided *p* = 0.0037]. Mean, standard error (SE), and 95% coefficient intervals (CI) are represented as green, pink, and black lines, respectively.

Simple linear regression analyses show that blood leukocyte DNAmAge (Figure 4A, *p* = 0.0026) and AgeAcc (Figure 4B, *p* = 0.0037), but not TL (Supplementary Figure 2, *p* = 0.4165), highly correlate with those of the IS cells.

### Determinants of Blood Leukocytes and IS Cells DNAmAge, AgeAcc, and TL

Multiple regression analyses of the influence of age, gender, ICS therapy, leukocytes (model a in Table 3)/neutrophils (10^3^/ml) (model b in Supplementary Table 7), and FEV_1_% on rising blood leukocyte TL, DNAmAge, and AgeAcc show that the main determinants are therapy without ICS (*p* = 0.0393 and *p* = 0.0538/*p* = 0.0285 and *p* = 0.0507) and decline in FEV_1_% (*p* = 0.0158 and *p* = 0.0533/*p* = 0.0154 and *p* = 0.0595) both when leukocytes and neutrophils (10^3^/ml) were considered. Considering blood leukocyte DNAmAge (model a in Table 3 and model b in Supplementary Table 7), age is confirmed as a determinant (*p* < 0.0001). None of the considered variables (age, gender, ICS therapy, leukocytes/neutrophils (10^3^/μl), and FEV_1_%) influence blood leukocyte TL (Table 3 and Supplementary Table 7).

**Table 3:** Multiple regression analysis (model a) of the influence of age, gender, ICS therapy, leukocytes (10^3^/ml), and FEV_1_% on blood leukocytes TL, DNAmAge, and AgeAcc.

	**Variables**	***B***	***R***	***t***	***p*-value**
***TL***					
	Age	b1 = −0.013682	*r* = −0.387393	*t* = −1.455632	*p* = 0.1712
	Gender (female)	b2 = −0.044425	*r* = −0.083364	*t* = −0.28979	*p* = 0.7769
	ICS therapy	b3 = 0.163057	*r* = 0.186039	*t* = 0.655908	*p* = 0.5243
	Leukocytes (10^3^/μl)	b4 = −0.041512	*r* = −0.3143	*t* = −1.146886	*p* = 0.2738
	FEV_1_%	b5 = −0.002728	*r* = −0.097522	*t* = −0.339446	*p* = 0.7401
***DNAmAge***					
	Age	b1 = 0.725707	*r* = 0.907802	*t* = 6.844848	***p*** **<** **0.0001**
	Gender (female)	b2 = 1.99263	*r* = 0.351855	*t* = 1.188675	*p* = 0.262
	ICS therapy	b3 = −6.191555	*r* = −0.599726	*t* = −2.370016	***p*** **=** **0.0393**
	Leukocytes (10^3^/μl)	b4 = 0.138452	*r* = 0.117325	*t* = 0.373595	*p* = 0.7165
	FEV_1_%	b5 = −0.224894	*r* = −0.675968	*t* = −2.900673	***p*** **=** **0.0158**
AgeAcc^*^					
	Gender (female)	b1 = 3.520267	*r* = 0.481361	*t* = 1.821398	*p* = 0.0958
	ICS therapy	b2 = −6.789407	*r* = −0.538208	*t* = −2.11795	***p*** **=** **0.0538**
	Leukocytes (10^3^/μl)	b3 = 0.437876	*r* = 0.291221	*t* = 1.009633	*p* = 0.3344
	FEV_1_%	b4 = −0.200247	*r* = −0.537241	*t* = −2.112601	***p*** **=** **0.0533**

* *The variable Age is not considered for AgeAcc because of its own definition*.

*Bold character is displayed only for significant values*.

Multiple linear regression analyses show that IS cell DNAmAge, AgeAcc, and TL are not related to any of the variables considered including age, gender, ICS therapy, neutrophil %, and FEV_1_% (Supplementary Table 8).

In a multiple linear regression analysis (Supplementary Table 9), in which also the type of inhalers was considered as independent variable, determinants that increase blood leukocyte DNAmAge are confirmed to be therapy without ICS, decline in FEV_1_%, and age (*p* = 0.0502, *p* = 0.0242, and *p* = 0.0001), but not the type of devices.

## Discussion

Aging is an individual and very complex process, and in the multifaceted framework of biological aging a variety of molecular, biochemical, and metabolic changes occur at the cellular level. In this study, we have determined the biological age of the IS cells and of peripheral blood leukocytes, by measuring the mitotic age (TL) and the non-mitotic epigenetic age (DNAmAge), in COPD patients as a positive paradigm of lung aging.

The main findings stemming from this work reveal that:

a) IS cells are biologically older than blood leukocytes as determined by DNAmAge, AgeAcc, and TL.
b) IS cell DNAmAge and AgeAcc, but not TL, highly correlate with those of blood leukocytes.

To the best of our knowledge, in this study for the first time, DNAmAge and AgeAcc are determined in IS cells and blood leukocytes in the same subject, showing that IS cells are in turn biologically older than blood leukocytes. Furthermore, the accelerated aging of the IS cells compared to that of blood leukocytes confirms that tissues and organs in our body may age at different rates within the same individuals, as we have already proved for donors' heart, where instead DNAmAge is consistently younger than that of blood leukocytes (13). DNA methylation is currently the most promising molecular marker for monitoring biological aging and predicting life expectancy (39). In humans, DNA methylation changes start early in life, as demonstrated by longitudinal studies of infants' blood (40, 41). Notably, these early epigenetic profiles continue to accumulate changes with the advancement of age as shown in twins that do not share the same habits and/or environments (42, 43), indicating aging-associated DNA methylation changes depending on environmental factors. The present study would suggest that airways cells are more exposed/receptive, than blood leukocytes, to track epigenetic changes. Studies have shown smoking-related methylation signatures in peripheral blood (44) and in IS (45, 46). In our study, the higher DNAmAge and AgeAcc of IS cells would suggest that age-related methylation genes would be the target of cigarette smoke injury.

Furthermore, increasing evidence suggests that there is acceleration in lung aging in COPD, with the accumulation of senescent cells in the lung (47). In particular, the chronic inflammation in COPD involves the recruitment of the major inflammatory cells including neutrophils, monocytes/macrophages, and eosinophils into the airways. These cells can be detected in induced sputum (48). The increased percentage of sputum neutrophils is a characteristic of COPD, and this neutrophilic inflammation is induced by cigarette smoke, bacteria, viruses, and oxidative stress resulting in the release of neutrophilic mediators (48). Comparing healthy subjects and COPD patients (matched for age, gender, and tobacco habits), Guiot et al. (49) found a quite different IS cellular profile. In COPD patients, the proportion of sputum neutrophils is generally higher than that of macrophages and linked to the disease severity. In healthy controls, the percentage of macrophages is instead higher than the percentage of neutrophils (49). Our study confirms these results on COPD patients by reporting an increased percentage of neutrophils compared to that of macrophages. Furthermore, while the differential cell count of IS affects biological age, that of blood does not. In particular, we observe an older DNAmAge in relation to an increase in neutrophils and to a decrease in macrophage percentage. This indicates that neutrophils in IS are biological older than macrophages and it may be ascribed to the timeline of leukocytes (neutrophils) bone marrow exit, extravasation, and tissue infiltration (48). It is possible that these innate immune cells present in the IS are activated and thus show accelerated aging. The younger DNAmAge of macrophages may be attributed to their renewal capacity in response to lung injury (50). Therefore, we cannot exclude that the increased biological aging in IS cells compared to that in blood leukocytes is also attributed to the migration of neutrophils into the lung tissue, suggesting that it may be in the context of COPD.

In line with results on epigenetic age, we found that IS cells' TL is shorter than that of blood leukocytes. The TL attrition found in the specimens derived from the target organ of the disease agrees with previous studies that reported shorter TL in lung tissues of COPD patients, associated with inflammation indicators (51). The TL shortening observed in IS cells of COPD patients is coherent with the hypothesis that an elevated oxidative stress and increased release of pro-inflammatory cytokines, probably derived from the past smoking history, lead to TL attrition (51). Cigarette smoke carries an abundance of well-known genotoxins including polycyclic aromatic hydrocarbons (PAHs), transition metals, and N-nitrosamines that directly, as catalysts for ROS production, and indirectly, through their metabolism, are important sources for ROS generation (52) and trigger the activation of proinflammatory responses in cells of the airway mucosa (53). These and our results disagree, however, with that of Saferali et al. (54) which reported longer TL in the DNA from lung biopsies compared to TL from blood of cancer patients. Divergent results may be ascribed to the disease, i.e., cancer, considered in the study of Saferali, and to the different cell types present in the lung biopsies. In the whole, our results suggest that heterogeneous aging among different tissues in the same disease may be in consequence of several factors, including differential exposure to environmental factors, the consequent oxidative stress, and inflammatory responses with a different tempo-spatial recruitment and distribution of cells.

We found a close nexus between DNAmAge and AgeAcc of the IS cells and blood leukocytes, advising that blood leukocytes, mirroring the respiratory tract status, could be a surrogate tissue for lung aging studies. Two recent large longitudinal studies from SAPALDIA and ECRHS cohorts (55) and KORA and NAS cohorts (29) found an increased blood leukocyte AgeAcc, estimated using the Horvath method (9), in relation to decline in FEV_1_, of COPD patients (29) and the general population (55). According to our results, these findings obtained in blood leukocytes would mirror what happens in the respiratory airways. Furthermore, our results could allow us to translate the investigation on biological aging aspects, linked to COPD, into the clinical practice through a simple blood sample. In a real clinical scenario, blood sample may be easily and quickly acquired when visiting COPD patients and sent to the laboratory for biological age analysis. However, some caution is mandatory since, according to our findings, the difference between DNAmAge in IS cells describing biological markers in lung as target tissue and blood leukocytes of COPD patients is almost 6 years. Further studies are therefore needed to optimize the use of blood as a surrogate indicator of IS cells' biological age in clinical practice.

While we confirmed that DNAmAge highly correlates with chronological age, we found that blood leukocyte AgeAcc significantly decreased with increased chronological age, indicating that the epigenetic clock in older patients reduces its speed of aging. Our results agree with the hypothesis that the ticking rate of the epigenetic clock slows down in later life, as proposed by Horvath (9).

Furthermore, we observed that blood leukocyte DNAmAge and AgeAcc significantly decrease (become younger) in COPD patients, with ICS therapy (triple therapy) and with enhancement in lung function (FEV_1_%). This also would imply that patients in dual therapy without ICS, because of a few exacerbations in the previous year and/or symptoms (according to the ABCD tool of GOLD guidelines 2021), are those that become older faster. By exploring the effects of systemic corticosteroid exposure, Wan et al. (56) found site-specific differences in blood DNA methylation of COPD patients. ICS anti-inflammatory therapy reduces pro-inflammatory mediator secretion from COPD alveolar macrophages exposed to microbial or oxidative stress triggers (57, 58). Since the inflammation represents a key aspect of aging, the anti-inflammatory role of ICS, together with their ability to determine alterations in the methylation profile, could explain the rejuvenating effect we found in COPD patients with ICS assumption in triple therapy. Our results are in line with those obtained from Lee et al. (59), reporting that asthmatic children exposed to air pollution who received ICS medication had longer TL than non-ICS users. With our findings, we strengthen the current literature that focuses on the role of age and aging-associated signaling pathways as well as their impact on current treatment strategies in the pathogenesis of COPD (60). However, future studies are warranted to determine the possible rejuvenating effect of ICS therapy we detected using biological age indicators.

Furthermore, we discovered that the severity of the disease measured by FEV_1_% is associated with the speeding up of blood leukocyte DNAmAge and AgeAcc. In line with our results, the longitudinal data from SAPALDIA and ECRHS cohorts (55) and KORA and NAS cohorts (29) report the association between blood AgeAcc [estimated using the Horvath method (9)] and lung function decline evaluated by FEV_1_ (55). FEV_1_% punctually expresses lung function as a percentage of the predicted normal value according to reference values based on age, height, weight, sex, and race for each subject using the European Community for Steel and Coal as reference values (34).

The current study presents weak points including the limited sample size of patients. However, the sample size estimation reveals that it is sufficient to obtain statistical significant results. The low yield of samples obtained from the IS technique is another weak point. The IS technique that allows collecting cells from airways, like biopsies and bronchoalveolar lavages, has, however, the advantages of being simple, well-tolerated, safe, reproducible, cost-effective, and noninvasive, making it one of the best alternatives of choice of airway sampling. From our clinical experience, it is a suitable procedure that could be applied in future studies and represents at the same time a strong point of this study. Previous work by Hosgood et al. (61), which measured TL and genetic variation in telomere maintenance genes in IS cells, even if not in the blood of the same subjects, confirms that this method was successful at collecting cells from the lung and bronchi. However, the IS technique must be performed under medical supervision and requires thorough instructions to patients from the specialized operator and the cooperation of patients, taking into account their medical condition, as respiratory efforts are needed. The last aspect explains the low success rate in our study. However, samples analyzed are adequate from the statistical point of view. The lack of an age-matched control group represents another limitation. Therefore, future research on COPD is mandatory and our future efforts will be directed to increasing the number of patients, also including a control group.

The strength of our study is that it supports the use of a validated noninvasive method of airway sampling for the analysis of biological age indicators in IS for future studies on biological aging of the lung. Furthermore, we showed that TL and DNAmAge in blood leukocytes correlate with those of IS cells. Determining the two most prominent biomarkers of biological age, DNAmAge and TL, with an almost totally automated workflow, is also a strong point of our study. We applied the method proposed by Zbieć-Piekarska et al. (11) to assess DNAmAge, on data from five CpG sites using the locus-specific technology pyrosequencing with some modification as described by Pavanello et al. (12, 13), which makes the technical analysis achievable in few hours. By using this process, we can perform the analyses in a standardized way while also reducing errors (see Supplementary Figure 3). It is noteworthy that pyrosequencing has the potential for multiplexing, which can simplify the protocol and reduce the cost of technical analysis. Although a real consensus on how to best detect and describe cellular senescence still remains to be achieved, we demonstrate the applicability of the IS cell mitotic age (TL) and non-mitotic epigenetic age (DNAmAge) analysis in molecular biological age profiling of COPD and relate them to the main clinical characteristics of the COPD disease. We add further information to what already exists on the analysis of IS.

In conclusion, new findings stemming from our study are that we detect a differential aging in the context of COPD by a direct quantitative comparison of cell aging in the airways with that in the more accessible peripheral blood leukocytes, providing additional knowledge which could offer certain potential translation into the disease management.

## Data Availability Statement

The raw data supporting the conclusions of this article will be made available by the authors, without undue reservation.

## Ethics Statement

The studies involving human participants were reviewed and approved by Ethics Committee of University-Hospital of Padova (code number 3843/AO/16 and 3054/AO/14). The patients/participants provided their written informed consent to participate in this study.

## Author Contributions

SP and MC: conceived and designed the study and administrative, technical, or material support (i.e., reporting or organizing data, constructing databases). GG, FL, and PM: patients' enrollment. GG, MC, and SP: provided the samples. MC and SP: performed the samples' analysis and wrote the paper. SP and MC: analyzed the data. All authors contributed to the article and approved the submitted version.

## Conflict of Interest

The authors declare that the research was conducted in the absence of any commercial or financial relationships that could be construed as a potential conflict of interest.

## Publisher's Note

All claims expressed in this article are solely those of the authors and do not necessarily represent those of their affiliated organizations, or those of the publisher, the editors and the reviewers. Any product that may be evaluated in this article, or claim that may be made by its manufacturer, is not guaranteed or endorsed by the publisher.

## Footnotes

^1^Available online at: https://www.who.int/news-room/fact-sheets/detail/chronic-obstructive-pulmonary-disease-(copd) (accessed June 01, 2021).

## Abbreviations

AgeAcc: age acceleration
COPD: chronic obstructive pulmonary disease
CRP: C-reactive protein
DNAmAge: DNA methylation age
FEV_1_: forced expiratory volume in one second
TL: telomere length.
