# Supplementary material for: DNA Methylation-Based Age Prediction and Telomere Length Reveal an Accelerated Aging in Induced Sputum Cells Compared to Blood Leukocytes: A Pilot Study in COPD Patients
Source: Front Med (Lausanne). 2021 Jul 23;8:690312. doi: 10.3389/fmed.2021.690312 (PMC8342924; doi:10.3389/fmed.2021.690312)
Supplement: Supplementary file 1 [file Data_Sheet_1.docx]

***Supplementary Material***

**1 SUPPLEMENTARY DATA**

**1.1 TL analysis**

TL was measured after DNA extraction from both whole blood leukocytes and induced sputum (IS) samples, by using quantitative Real-Time PCR as previously described (1, 2). This assay measures relative TL in genomic DNA by determining the ratio of telomere repeat copy number (T) to single nuclear copy gene (S), i.e. the T/S ratio, in a given sample relative to reference DNA. The single-copy gene used was human (beta) globin (hbg). As reference DNA, we used a pool of DNA from the study population. Two different pool of DNA were made for TL analyses on DNA extracted from whole blood leukocytes and induced sputum samples. A fresh seven points standard curve from the pool, ranging from 40 to 0.625 ng/µl (serial dilutions 1:2), was included in every “T” and “S” PCR run, against a negative control (water). In brief, Qiagility (QIAGEN, Milano, Italy) that enables a high-precision PCR set up, was used for transferring 10 µl of reaction mix and 5 µl of DNA (5 ng/µl) in a 96-well plate. In total, 25 ng of DNA sample was added to each reaction, and each sample was run in triplicate. All PCR reactions were performed on a SteponePlus Real-Time PCR System (Applied Biosystems). The average of the three T measurements was divided by the average of the three S measurements to calculate the average T:S ratio, i.e. the relative telomere length. A measure was considered acceptable if the standard deviation (SD) among triplicate measures was <0.25. The coefficient of variation for the average T:S ratio of samples analyzed over three consecutive days was 10%, which was similar to the reproducibility originally reported for this method (3).

**1.2 DNAmAge analysis**

DNAmAge was determined by analysis the methylation levels from selected markers using bisulfite conversion and Pyrosequencing® methodology as previously reported (4, 5). This method is based on determination of the methylation level of a set of five markers (ELOVL2, C1orf132, KLF14, TRIM59 and FHL2) in genomic DNA, as described by Zbieć-Piekarska et al. (6) with some modifications based on the fact that the method was almost completely automated using the PyroMark Q48 Autoprep (QIAGEN, Milano, Italy). Briefly, 1 μg DNA was submitted to bisulfite conversion: unmethylated cytosines in extracted DNA were converted to uracil using Epitect Fast® DNA Bisulfite Kit (QIAGEN, Milano, Italy) following the manufacturer’s instructions. An aliquot of template DNA was used for PCR amplification of selected markers using PCR primers included in the AgePlex Mono kit (Biovectis, Warszawa, Poland). PCR reactions were performed in 25 μL, comprising 0.2 μM of each primers, 20 ng of template DNA, and PyroMark PCR Master Mix holding HotStarTaq DNA Polymerase, 1X PyroMark PCR Buffer and dNTPs. The amplification plan involved a preliminary denaturation step at 95 °C for 10 min, followed by 40–45 cycles of denaturation (94 °C for 30 s), annealing (56 °C for 60 s) and extension (72 °C for 90 s), and a final extension of 72 °C for 10 min. Each PCR amplification contained negative PCR controls. In total, 10 µL of PCR product was used for each pyrosequencing primer (2 µL) contained in AgePlex Mono kit (Biovectis, Warszawa, Poland) and loaded into a 48 well-plate (Pyromark Q48 Discs, QIAGEN, Milano, Italy). Details on PCR and sequencing primer sequences and the sequences analyzed are given in Table S1. Pyrosequencing was performed on a Pyromark Q48 Autoprep instrument (QIAGEN, Milano, Italy) using Pyromark Q48 Advanced Reagents (QIAGEN, Milano, Italy) according to the manufacturer’s instructions. The resulting Pyrograms® generated by the instrument were automatically analyzed using Pyromark Q48 Autoprep Software (QIAGEN, Milano, Italy). The level of methylation was expressed as a percentage of methylated cytosines at the 5 CpG sites considered. The methylation percentages were inserted in an online calculator system accessible at *www.agecalculator.ies.krakow.pl*, for estimation of biological age from DNA methylation analysis. The equation corresponds to a previously developed age prediction model (6). All samples were analyzed 3 times for each marker to verify the reproducibility of our results, and their averages were utilized in the statistical testing. All samples were analyzed on two different days, and the coefficient of variation (CV) for replicate pyrosequencing runs was 0.5%.

**References**

1. Pavanello, S., Stendardo, M., Mastrangelo, G., Bonci, M., Bottazzi, B., Campisi, M., et al. (2017). Inflammatory long pentraxin 3 is associated with leukocyte telomere length in night-shift workers. Front. Immunol. 8: 516. doi: 10.3389/fimmu.2017.00516.
2. Pavanello, S., Angelici, L., Hoxha, M., Cantone, L., Campisi, M., Tirelli, A.S., et al. (2018). Sterol 27-hydroxylase polymorphism significantly associates with shorter telomere, higher cardiovascular and type-2 diabetes risk in obese subjects. Front. Endocrinol. (Lausanne) 9: 309. doi: 10.3389/fendo.2018.00309.
3. Cawthon, R. M. (2002). Telomere measurement by quantitative PCR. Nucleic Acids Res. 30: 47. doi:10.1093/nar/30.10.e47.
4. Pavanello, S., Campisi, M., Tona, F., Dal Lin, C., Iliceto, S. (2019). Exploring epigenetic age in response to intensive relaxing training: a pilot study to slow down biological age. Int. J. Environ. Res. Public. Health. 16: 3074. doi: 10.3390/ijerph16173074.
5. Pavanello, S., Campisi, M., Fabozzo, A., Cibin, G., Tarzia, V., Toscano, G., et al. (2020). The biological age of the heart is consistently younger than chronological age. Sci Rep. 10: 10752. doi: 10.1038/s41598-020-67622-1.
6. Zbieć-Piekarska, R., Spólnicka, M., Kupie, T., Parys-Proszek, A., Makowska, Ż., Pałeczka, A., et al. (2015). Development of a forensically useful age prediction method based on DNA methylation analysis. Forensic Sci. Int. Genet. 17: 173-9. doi: 10.1016/j.fsigen.2015.05.001.

**SUPPLEMENTARY TABLES AND FIGURES**

**2.1 Tables**

**Table S1. PCR and Pyrosequencing primer sequences, and sequences to analyse.**

| **Marker** | **Primers** | **Primer sequence** | **Sequence to analyse** |
| --- | --- | --- | --- |
| ELOVL2 | Forward ^B^ | AGGGGAGTAGGGTAAGTGAGG | CCRTAAACRTTAAACCRCCRCRCRAAACCRAC |
|  | Reverse | AACAAAACCATTTCCCCCTAATAT |  |
|  | Sequencing | ACAACCAATAAATATTCCTAAAACT |  |
| C1orf132 | Forward ^B^ | GTAAATATATAAGTGGGGGAAGAAGGG | AAATCTACRCAAACRACRATAAATAATCC |
|  | Reverse | TTAATAAAACCAAATTCTAAAACATTC |  |
|  | Sequencing | CACCTTACCACCAAACCAAAATTT |  |
| TRIM59 | Forward | TATAGGTGGTTTGGGGGAGAG | GGTTTGGYGY GGGAYGAGGYGAAGYGTYGG TGGTYGAYGG TTTTTGAGGA ATTATTTTTT ATTT |
|  | Reverse ^B^ | AAAAAACACTACCCTCCACAACATAAC |  |
|  | Sequencing | TTGGGGGAGAGGTTG |  |
| KLF14 | Forward | GGTTTTTAGGTTAAGTTATGTTTAATAGT | TYGYGTTTTTTTTTTTGTYGGYGAGTTAGGTA ATGGTAATAGAG |
|  | Reverse ^B^ | ACTACTACAACCCAAAAATTCC |  |
|  | Sequencing | ATAGTTTTAGAAATTATTTTGTTT |  |
| FHL2 | Forward | TGTTTTTAGGGTTTTGGGAGTATAG | AGTTATYGGG AGYGTYGTTT TYGGYGTGGG TTTTYGGGYG YGAGTTTYGG AYGAGGTTTG GG |
|  | Reverse ^B^ | ACACCTCCTAAAACTTCTCCAATCTCC |  |
|  | Sequencing | GGTTTTGGGAGTATAGT |  |

**Table S2.** DNAmAge and AgeAcc of COPD patients’ IS cells and blood leukocytes.

|  | **Chronological age (years)** | **DNAmAge(years)** | | | **AgeAcc (DNAmAge-chronological age)** | | |
| --- | --- | --- | --- | --- | --- | --- | --- |
|  |  | IS cells | Blood leukocytes | Blood leukocytes | IS cells | Blood leukocytes | Blood leukocytes |
| N | 16 | 7 | 7 | 16 | 7 | 7 | 16 |
| Mean±SD | 74±7 | 67.4±5.80^§^ | 61.6±5.40^§^ | 63.3±5.60 | -4.5±5.02^§†^ | -10.8±3.50^§^ | -10.3±3.63^†^ |

^§^ DNAmAge Paired t tests on n=7: IS cells versus blood leukocytes p=0.0003

^§^ AgeAcc Paired t tests on n=7: IS cells versus blood leukocytes p=0.0003

^†^ AgeAcc IS cells versus blood leukocytes Mann-Whitney U test Two sided p =0.0156

**Table S3**. Telomere length of COPD patients’ IS cells and blood leukocytes.

|  | **Chronological age (years)** | **Telomere length (T/S)** | | | |
| --- | --- | --- | --- | --- | --- |
|  |  | IS cells | Blood leukocytes | Blood leukocytes |  |
| N | 18 | 8 | 8 | 18 |  |
| Mean±SD | 72±8 | 1.05±0.35^§†^ | 1.48±0.21^§^ | 1.47±0.26^†^ |  |

^§^ Paired t tests on n=8: IS cells versus blood leukocytes p= 0.0341

^†^Mann-Whitney U test; Two sided p=0.0133

Table S4. Leukocytes and differential cell count (mean ± standard deviation) in blood samples (n=18) from COPD patients and correlations with blood leukocytes DNAmAge, AgeAcc and TL.

| Variables | Blood samples | DNAmAge | | | AgeAcc | | | TL | | |
| --- | --- | --- | --- | --- | --- | --- | --- | --- | --- | --- |
|  | 10^3^/ml | *b* | *r* | *p-Value* | *b* | *r* | *p-Value* | *b* | *r* | *p-Value* |
| Leukocytes | 6.5 ± 1.9 | -0.208652 | 0.077895 | 0.7743 | 0.282457 | 0.162366 | 0.548 | -0.018003 | 0.139166 | 0.5818 |
| Neutrophils | 3.99 ± 1.4 | 0.369117 | 0.0968 | 0.7214 | 0.515955 | 0.208343 | 0.4387 | -0.00577 | 0.030924 | 0.903 |
| Lymphocytes | 1.6 ± 0.6 | -3.802914 | 0.401409 | 0.1233 | 0.757141 | 0.123056 | 0.6498 | -0.176601 | 0.369892 | 0.1308 |
| Monocytes | 0.6 ± 0.2 | -6.633726 | 0.211149 | 0.4325 | -6.644239 | 0.325637 | 0.2184 | -0.080144 | 0.052819 | 0.8351 |
| Eosinophils | 0.2 ± 0.3 | 1.274415 | 0.073892 | 0.7856 | 1.853407 | 0.165469 | 0.5403 | -0.088208 | 0.106798 | 0.6732 |
| Basophils | 0.04 ± 0.04 | -6.967213 | 0.025115 | 0.9264 | 18.032787 | 0.100091 | 0.7123 | 2.329327 | 0.396906 | 0.1029 |

Table S5. Influence of cigarette smoking (pack years) on DNAmAge, AgeAcc and TL of blood leukocytes in COPD patients.

| Variables | DNAmAge | | | AgeAcc | | | TL | | |
| --- | --- | --- | --- | --- | --- | --- | --- | --- | --- |
|  | *b* | *r* | *p-Value* | *b* | *r* | *p-Value* | *b* | *r* | *p-Value* |
| Pack years | -0.060946 | 0.32614 | 0.21723 | -0.1112626 | 0.101321 | 0.711344 | -0.001003 | 0.070123 | 0.788232 |

Table S6. Differential cell count (mean ± standard deviation) in induced sputum samples (n=8) from COPD patients and correlations with DNAmAge, AgeAcc and TL.

| Variables | Induced sputum samples | DNAmAge | | | AgeAcc | | | TL | | |
| --- | --- | --- | --- | --- | --- | --- | --- | --- | --- | --- |
|  | % | *b* | *r* | *p-Value* | *b* | *r* | *p-Value* | *b* | *r* | *p-Value* |
| Macrophages* | 25.19±16.81 | -0.269429 | 0.794108 | **0.033** | -0.12999 | 0.464252 | 0.294 | 0.005424 | 0.26084 | 0.5327 |
| Neutrophils* | 68.06±27.03 | 0.184837 | 0.869521 | **0.011** | 0.107783 | 0.6144 | 0.1421 | 0.000779 | 0.060204 | 0.8874 |
| Eosinophils* | 6.75±15.70 | -0.231865 | 0.636945 | 0.1239 | -0.166461 | 0.5541 | 0.1968 | -0.008525 | 0.382861 | 0.3492 |
| Lymphocytes* | 0 | / | / | / | / | / | / | / | / | / |

* The differential cell count (%) is normalized on percentage of squamous cells equal to 4.23±7,69 percent, that is acceptable since it is <20%.

Bold character is displayed only for significant values.

**Table S7.** Multiple regression analyses (model b) of the influence of age, gender, ICS therapy, neutrophils (10^3^/ml) and FEV_1_% on blood leukocytes TL, DNAmAge and AgeAcc.

|  | **Variables** | **b** | **r** | **t** | **p-Value** |  |
| --- | --- | --- | --- | --- | --- | --- |
|  | Age | b1 = -0.002195 | r = -0.072917 | t = -0.242483 | p = 0.8129 |  |
| ***TL*** | Gender (female) | b2 = -0.097448 | r = -0.217252 | t = -0.738176 | p = 0.4759 |  |
|  | ICS therapy | b3 = 0.229537 | r = 0.30522 | t = 1.063024 | p = 0.3106 |  |
|  | Neutrophils (10^3^/µl) | b4 = -0.064153 | r = -0.383428 | t = -1.376923 | p = 0.1959 |  |
|  | FEV_1_% | b5 = -0.003794 | r = -0.164426 | t = -0.552864 | p = 0.5914 |  |
| ***DNAmAge*** | **Variables** | **b** | **r** | **t** | **p-Value** |  |
|  | Age | b1 = 0.728536 | r = 0.918393 | t = 7.339978 | **p < 0.0001** |  |
|  | Gender (female) | b2 = 2.258686 | r = 0.411894 | t = 1.429409 | p = 0.1834 |  |
|  | ICS therapy | b3 = -6.580395 | r = -0.628937 | t = -2.558181 | **p = 0.0285** |  |
|  | Neutrophils (10^3^/µl) | b4 = 0.458176 | r = 0.267246 | t = 0.877003 | p = 0.4011 |  |
|  | FEV_1_% | b5 = -0.21939 | r = -0.678127 | t = -2.917802 | **p = 0.0154** |  |
| ***AgeAcc**** | **Variables** | **b** | **r** | **t** | **p-Value** |  |
|  | Gender (female) | b1 = 3.530659 | r = 0.488065 | t = 1.854622 | p = 0.0906 |  |
|  | ICS therapy | b2 = -7.09216 | r = -0.551543 | t = -2.192973 | **p = 0.0507** |  |
|  | Neutrophils (10^3^/µl) | b3 = 0.70766 | r = 0.312552 | t = 1.091289 | p = 0.2985 |  |
|  | FEV_1_% | b4 = -0.198071 | r = -0.535142 | t = -2.101022 | **p = 0.0595** |  |

*The variable Age is not considered for AgeAcc because of its own definition.

Bold character is displayed only for significant values.

**Table S8.** Multiple regression analyses of the influence of age, gender, ICS therapy, neutrophils (%) and FEV_1_% on IS cells TL, DNAmAge and AgeAcc.

|  | **Variables** | **b** | **r** | **t** | **p-Value** |  |
| --- | --- | --- | --- | --- | --- | --- |
|  | Age | b1 = 0.023191 | r = 0,795448 | t = 1,312574 | p = 0.4145 |  |
| ***TL*** | Gender (female) | b2 = 0.832905 | r = 0.939607 | t = 2.745347 | p = 0.2224 |  |
|  | ICS therapy | b3 = 0.170976 | r = 0.659159 | t = 0.876536 | p = 0.5418 |  |
|  | Neutrophils (%)** | b4 = -0.006438 | r = -0.833877 | t = -1.510781 | p = 0.3722 |  |
|  | FEV_1_% | b5 = 0.043988 | r = 0.921654 | t = 2.375321 | p = 0.2537 |  |
| ***DNAmAge*** | **Variables** | **b** | **r** | **t** | **p-Value** |  |
|  | Age | b1 = 0.386295 | r = 0.969092 | t = 3.92821 | p = 0.1587 |  |
|  | Gender (female) | b2 = -1.810935 | r = -0.731373 | t = -1.072428 | p = 0.4778 |  |
|  | ICS therapy | b3 = 3.628364 | r = 0.958032 | t = 3.34203 | p = 0.1851 |  |
|  | Neutrophils (%)** | b4 = 0.156678 | r = 0.988733 | t = 6.605323 | p = 0.0957 |  |
|  | FEV_1_% | b5 = 0.320688 | r = 0.952033 | t = 3.111261 | p = 0.198 |  |
| ***AgeAcc**** | **Variables** | **b** | **r** | **t** | **p-Value** |  |
|  | Gender (female) | b1 = 1.869153 | r = 0.154032 | t = 0.220465 | p = 0.846 |  |
|  | ICS therapy | b2 = -4.391965 | r = -0.316515 | t = -0.47188 | p = 0.6835 |  |
|  | Neutrophils (%)** | b3 = 0.101017 | r = 0.561562 | t = 0.959796 | P = 0.4384 |  |
|  | FEV_1_% | b4 = 0.000559 | r = 0.001171 | t = 0.001657 | p = 0.9988 |  |

*The variable Age is not considered for AgeAcc because of its own definition.

** The neutrophils count (%) is normalized on percentage of squamous cells in IS equal to 4.23±7,69 percent, that is acceptable since it is <20%.

Table S9. Multiple regression analyses of the influence of age, gender, ICS therapy, type of inhalers leukocytes (10^3^/ml) and FEV_1_% on blood leukocytes DNAmAge.

| ***DNAmAge*** | **Variables** | **b** | **r** | **t** | **p-Value** |
| --- | --- | --- | --- | --- | --- |
|  | Age | b1 = 0.723043 | r = 0.906097 | t = 6.425145 | **p = 0.0001** |
|  | Gender (female) | b2 = 1.904646 | r = 0.327724 | t = 1.040642 | p = 0.3252 |
|  | ICS therapy | b3 = -6.227133 | r = -0.601627 | t = -2.259557 | **p = 0.0502** |
|  | Type of inhalers | b4 = -0.276655 | r = -0.059921 | t = -0.180087 | p = 0.8611 |
|  | Neutrophils (10^3^/µl) | b5 = 0.13748 | r = 0.116709 | t = 0.352535 | p = 0.7326 |
|  | FEV_1_% | b6 = -0.228977 | r = -0.669558 | t = -2.704342 | **p = 0.0242** |

Bold character is displayed only for significant values.

**2.2 Figures**

| **(A)**   | **(B)**   |
| --- | --- |
| **(C)**   | **(D)**   |

**Figure S1. Correlation curves between DNAmAge (A), AgeAcc (B), TL (C) of induced sputum cells and chronological age of n=7 COPD patients, as well as between blood leukocytes** **TL (D) and chronological age of n=18 COPD patients.**

In (A) and in (B), simple linear regression plots showing the correlation between DNAmAge and AgeAcc of the induced sputum cells and chronological age (Correlation coefficient (r)=0.654862, Two sided p=0.1104 for DNAmAge; and r=-0.401708, Two sided p=0.3717 for AgeAcc ); while in (C), simple linear regression plot showing the correlation between TL of the induced sputum cells and chronological age (r=0.30671, Two sided p=0.460). In (D) simple linear regression plot showing the correlation between TL of the blood leucocytes and chronological age (r=-0.27439, Two sided p=0.2705).

Mean, Standard Error (SE) and 95% coefficient intervals (CI) are represented as green, pink and black lines, respectively.

**Figure S2. Correlation curves between TL of blood leukocytes and induced sputum cells in n=8 COPD patients.**

Simple linear regression plot shows the correlation between TL of blood leukocytes and induced sputum cells (r=-0.33554; Two sided p=0.4165).

Mean, Standard Error (SE) and 95% coefficient intervals (CI) are represented as green, pink and black lines, respectively.


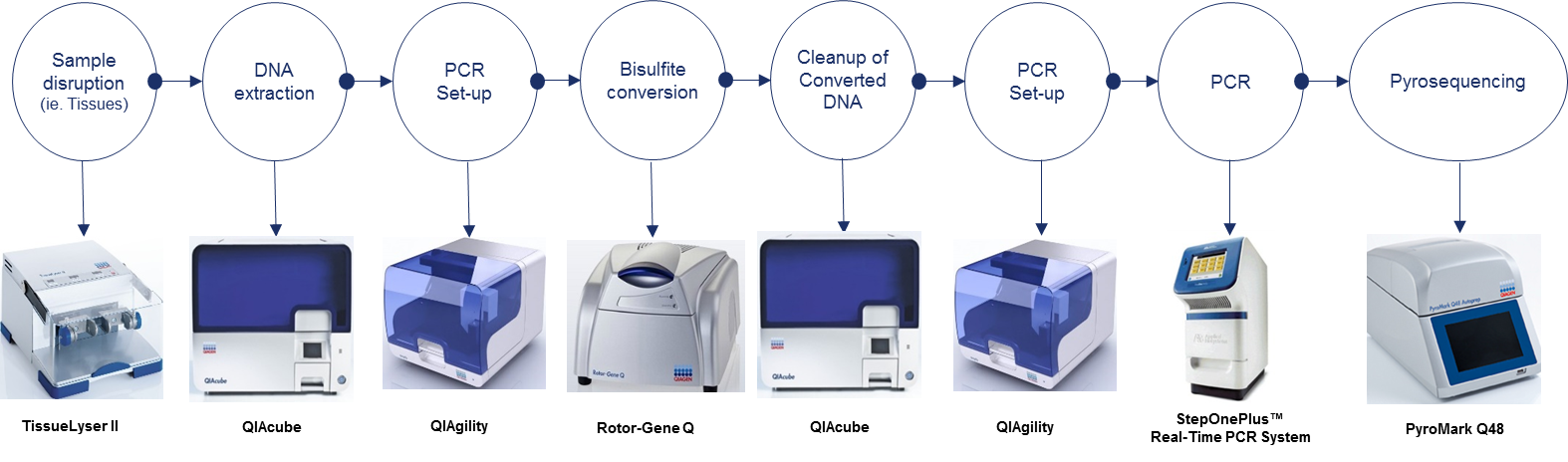


Figure S3. Seamless Workflow from sample to data interpretation. In brief, DNA extraction is accomplished on QIAcube workstation (QIAGEN). For DNAmAge analysis, the genomic DNA extracted is submitted to bisulfite conversion on Rotor-Gene Q (QIAGEN), a real-time PCR cycler, and all reactions are prepared on QIAgility (QIAGEN), which is an instrument that enables automated high-precision setup of PCR experiments. The clean-up of converted DNA is performed on the QIAcube (QIAGEN). An aliquot of template DNA is used for PCR amplification of selected markers on StepOnePlus™ Real-Time PCR System (Applied Biosystems™) and the PCR setup is automated performed on Qiagility workstation (QIAGEN). Pyrosequencing for DNA methylation analysis is carried out on PyroMark Q48 Autoprep (QIAGEN). The resulting Pyrograms® are automatically analyzed using PyroMark Q48 Autoprep Software. For TL analysis, an aliquot of genomic DNA extracted is used for Real-Time PCR on StepOnePlus™ Real-Time PCR System (Applied Biosystems™). All reactions and the PCR setup are performed on Qiagility (QIAGEN). The results are analyzed using StepOne Software 2.3.
